# Contact Days Associated With Cancer Treatments in the CCTG LY.12 Trial

**DOI:** 10.1093/oncolo/oyad128

**Published:** 2023-05-25

**Authors:** Arjun Gupta, Annette E Hay, Michael Crump, Marina S Djurfeldt, Liting Zhu, Matthew C Cheung, Lois E Shepherd, Bingshu E Chen, Christopher M Booth

**Affiliations:** Division of Hematology, Oncology, and Transplantation, University of Minnesota, Minneapolis, MN, USA; Canadian Cancer Trials Group, Kingston, ON, Canada; Department of Medicine, Queen’s University, Kingston, ON, Canada; Canadian Cancer Trials Group, Kingston, ON, Canada; Division of Hematology, Princess Margaret Hospital and University of Toronto, Toronto, ON, Canada; Canadian Cancer Trials Group, Kingston, ON, Canada; Canadian Cancer Trials Group, Kingston, ON, Canada; Canadian Cancer Trials Group, Kingston, ON, Canada; Division of Hematology, Odette Cancer Centre, Toronto, ON, Canada; Canadian Cancer Trials Group, Kingston, ON, Canada; Canadian Cancer Trials Group, Kingston, ON, Canada; Department of Oncology, Queen’s University, Kingston, ON, Canada

**Keywords:** contact days, home days, time toxicity, clinical trial, lymphoma, decision making

## Abstract

**Background:**

When cancer treatments have similar oncologic outcomes, the number of days with in-person healthcare contact (“”contact days’’) can help contextualize expected time use with each treatment. We assessed contact days in a completed randomized clinical trial.

**Patients and Methods:**

We conducted a secondary analysis of the CCTG LY.12 RCT that evaluated 2-3 cycles of gemcitabine, dexamethasone, and cisplatin (GDP) vs. dexamethasone, cytarabine, and cisplatin (DHAP) in 619 patients with relapsed/refractory lymphoma prior to stem cell transplant. Primary analyses reported similar response rates and survival. We calculated patient-level “contact days” by analyzing trial forms. The study period was from assignment to progression or transplant. Days without healthcare contact were considered “home days’’. We compared measures of contact days across arms.

**Results:**

The study period was longer in the GDP arm (median 50, vs. 47 days, *P = .*007). Contact days were comparable in both arms (median 18 vs 19, *P* = 0.79), but home days were higher in the GDP arm (median 33 vs 28, *P < .*001). The proportion of contact days was lower in the GDP arm (34%, vs. 38%, *P = .*009). The GDP arm experienced more contact days related to planned outpatient chemotherapy (median, 10 vs. 8 days), but the DHAP arm experienced many more inpatient contact days (median, 11 vs. 0 days).

**Conclusions:**

Measures of time use, such as contact days, can be extracted from RCTs. In LY.12, despite comparable oncologic outcomes, GDP was associated with fewer contact days. Such information can guide decision-making for patients with hematological cancers, who already face significant healthcare contact.

## Teaser

When cancer treatments have similar oncologic outcomes, the number of days with in-person healthcare contact (“contact days’’) can help contextualize expected time use with each treatment. This secondary analysis of the CCTG LY.12 trial analyzed trial forms to assess days with and without health care contact.

**Implications of Practice**

We extracted a patient-centered and pragmatic measure of the time spent with healthcare contact from a completed clinical trial. We identified days with in-person healthcare contact (“”contact days’’) and days spent at home (“”home days’’). In the LY.12 trial, we demonstrate that although 2 different chemotherapy regimens were associated with similar short- and long-term oncologic outcomes, one chemotherapy regimen was associated with more contact days and many more hospitalized days. Oncology clinical trial reports that present such data alongside traditional efficacy endpoints can help patients understand expected time use patterns and guide patient-oncologist decision-making regarding pursuing treatment approaches.

## Introduction

The time burdens imposed by cancer care are increasingly recognized, especially among persons with advanced solid tumors where the absolute survival benefit offered by a treatment may be small.^1-4^ Oncology clinical trials to date do not typically report the time requirements of treatment, making it difficult for oncologists to guide patients regarding the relative time spent with different treatment approaches. We initially termed these time costs as “’time toxicity”’ to encourage the oncology community to consider these as an adverse event of treatment.^1,5^ It is difficult, although, to accurately ascertain if time spent in a specific healthcare activity is necessary (or unnecessary), goal concordant (or discordant), or ­cancer-related (or treatment-related).^6^ What may be “’time toxic”’ to one patient may not be to another, and patients may derive psychosocial benefits from healthcare contact, beyond what is easily measurable (eg, feeling of comfort, security, etc.). Additionally, healthcare contact also represents access to healthcare, and we must be wary in interpreting less time in healthcare as universally good.^6^ Thus, it is important to be objective when measuring and reporting time spent with healthcare contact.

To optimize patient centeredness (since seemingly short days can turn into all-day affairs) and practicality (dichotomous and easily determined outcome), our proposed measure of this time spent considers a day with in-person healthcare system contact as a healthcare contact day, or a “”contact day’’. We have demonstrated that in a clinical trial in patients with advanced colorectal cancer, the additional 6 weeks of increased overall survival with the experimental agent was offset by an equal number of contact days.^5^ The number of days without in-person healthcare contact, what we term as “”home days’’, were similar between arms, and the proportion of contact days was 18% in the drug arm, and 6% in the supportive care alone arm.^5^ Such data, presented alongside traditional survival endpoints can provide for richer, more informed decision making.

While a majority of our initial work has focused on persons with advanced solid tumors, burdens of care are relevant to people with hematological malignancies. Prior work in hematology malignancies has largely focused on inpatient days, or nights spent away from home, and has not consistently accounted for outpatient visits.^7,8^ This follows similar approaches in the surgical, critical, and organ transplantation disciplines where a one-time inpatient intervention is followed by an intensive but shorter assessment period (eg, 90 days post-intervention).^1^ For example, studies have evaluated “”days alive and out of the hospital’’ in the first 100 days after stem cell transplantation.^7,8^ While foundational, this work by definition does not consider patient and care partner time use related to outpatient visits. Our current measure, considering all in-person healthcare contact, is more comprehensive. Details on specific sources of contact (eg, outpatient, inpatient) can provide additional insight.

As with solid tumors, different cancer treatment approaches for hematological malignancies may have similar or marginally different oncologic outcomes. In such cases, their associated contact days can aid patient-oncologist decision making regarding which treatment approach to pursue. We conducted a secondary analysis of a completed randomized controlled trial in patients with relapsed/ refractory lymphoma to assess the feasibility of extracting and comparing contact days and home days between arms.

## Methods

### Study Background, Procedures, and Initial Efficacy/Safety Data

We conducted a secondary analysis of the Canadian Cancer Trials Group LY.12 randomized clinical trial (ClinicalTrials.gov Identifier: NCT00078949). LY.12 recruited participants in Canada, Italy, US, and Australia during 2003-2011. Eligible adults had an aggressive histology relapsed/ refractory non-Hodgkin’s lymphoma that had relapsed after one ­anthracycline-containing regimen. Patients were randomized 1:1 to receive 2-3 outpatient cycles of gemcitabine, dexamethasone, and cisplatin (GDP) vs. dexamethasone, cytarabine, and cisplatin (DHAP) prior to autologous stem cell transplant. The third cycle was permitted if response to 2 cycles was insufficient. A protocol amendment in 2005 allowed patients with B-cell malignancies in both arms to additionally receive rituximab. The therapies were delivered in 21-day cycles, as presented in Table 1. GDP was administered in the outpatient setting, and each cycle required at least 2 outpatient visits to the infusion center, on days 1 and 8. DHAP was administered inpatient, with each cycle often requiring a 3-day/2-night hospitalization. Treatment could be delayed, and granulocyte colony-stimulating factor administration could be added for neutropenia, increasing potential ­treatment-related visits. Patients in both arms were evaluated in clinic on day 1 of each cycle, completed bloodwork on days 1 and 8 of each cycle, and underwent imaging after cycle 2 (and after cycle 3 if administered).

**Table 1.** Drugs and schedule of chemotherapy administration in both arms of the LY.12 trial.

	Gemcitabine	Dexamethasone	Cisplatin	Cytarabine	Rituximab
**GDP (or R-GDP)**	Intravenous over 30-minutes, day 1 and 8	Oral, days 1-4	Intravenous over 60-minutes, day 1	—	Intravenous over 3-6 hours, day 1
**DHAP (or R-DHAP)**	—	Oral, days 1-4	Intravenous infusion over 24 hours, day 1	Intravenous over 3 hours, for 2 doses 12 hours apart on day 2	Intravenous over 3-6 hours, day 1

Each regimen was delivered in 21-day cycles. Dose adjustments and deferments, and addition of growth factor support was allowed.

The primary trial endpoint was response rate with a ­non-inferiority design. Secondary endpoints included event-free survival, overall survival, rates of stem-cell mobilization, adverse events, quality-of-life, and healthcare utilization.

A total of 619 patients were randomized. Initial primary results published in 2014 reported similar response rates (44%-45%) between arms, confirming the ­non-inferiority of GDP. Transplantation rates (49%-52%), 4-year ­event-free-survival (26%), and 4-year overall survival (39%) were also similar between arms.^9^ Patients in the GDP arm suffered less severe toxicity, were hospitalized less often, and experienced less deterioration in quality of life.^9^ Secondary analyses had noted that GDP was associated with less healthcare spending.^10,11^

### Study Procedures for the Current Analyses

The study period for the current analysis was the date of assignment to the date of progression or the day before stem cell transplant (whichever was earlier), as in prior work.^10,11^ We chose this start date since protocol treatment had to be initiated within 5 days of randomization per trial policy. We chose this end date because then the study period reflected the specific time impacts of GDP vs. DHAP, without being affected by later stem cell transplantation.

We considered any day with any amount of in-person healthcare system contact as a “”contact day’’.^1^ Thus, an outpatient visit for bloodwork, for chemotherapy, and to see a clinician were treated the same, as a visit to an urgent care, or a night in the hospital or a rehabilitation facility. Multiple appointments on the same day only counted as a single contact day. A day without in-person healthcare contact was considered a “”home day’’. Thus, for each individual patient, the sum of contact days and home days was the study period in days. We further extracted and analyzed specific sources of contact days (eg,, for planned chemotherapy, for bloodwork, hospitalized, etc.). We calculated patient-level contact days by analyzing relevant case report and resource utilization forms collected for each participant. This was possible because the trial included a pre-planned economic analysis and collected health care utilization data. This included planned healthcare contact for chemotherapy utilization, supportive care interventions, outpatient labs and other diagnostic tests, outpatient clinic visits, outpatient infusion center visits, and inpatient care. As an example, the “”chemotherapy report form’’ was completed for each cycle of chemotherapy, and collected treatment data, toxicity data, response status, and healthcare utilization during each 3-week period. It collected both ­protocol-related and unplanned healthcare utilization such as outpatient visits and procedures (eg, transfusions), emergency room visits, and hospitalizations, along with the date(s) of healthcare contact.

### Statistical Analysis

We compared proportions and medians of time measures across arms by a χ^2^ test and Wilcoxon test. All randomized patients were included in the analyses on the base of ­intention-to-treat. All reported *P* values are 2-sided and were not adjusted for multiple testing. The study was conducted according to the guidelines of the Declaration of Helsinki, and approved by the Institutional Review Board (or Ethics Committee) of University Health Network. All patients provided written informed consent before participation.

## Results

Six-hundred and 10 of the 619 (98.5%) trial participants had data on contact days available and were included in the current analyses. The median age was 55 years, 61% of participants were men, and 86% had an ECOG performance status of 0-1. Detailed baseline characteristics are available in prior publications.^9,12^

Data on contact days and their sources, and home days are presented in Table 2. The median study duration was approximately 7 weeks in both arms. It was slightly longer in the GDP arm (median, 50 days) than in the DHAP arm (median, 47 days). This difference was statistically significant (*P* = .007). Median contact days were comparable in both arms (18 vs 19 days, *P* = .79), but median home days were higher in the GDP arm (33 vs 28 days, *P* < .001). The proportion of contact days (contact days divided by study duration) was lower in the GDP arm (34%, vs. 38%, *P* = .009). The GDP arm experienced more contact related to planned outpatient chemotherapy (median, 10 vs. 8 days), but the DHAP arm experienced many more contact days hospitalized (median, 11 vs. 0 days).

**Table 2.** Measures of time use in LY.12 for the overall study population, data are median (interquartile range).

Time use measure	Gemcitabine, dexamethasone, and cisplatin *N* = 306	Dexamethasone, cytarabine, and cisplatin *N* = 304	*P*-value
Study duration, days	50 (43, 64)	47 (42, 62)	.007
Contact days	18 (15, 23)	19 (14, 26)	.79
Home days	33 (25, 43)	28 (20, 39)	<.001
Proportion of contact days (contact days/study duration), %	34 (29, 44)	38 (28, 50)	.009
*Specific sources of contact days (not mutually exclusive)*			
Chemotherapy (outpatient or inpatient)	10 (10, 14)	8 (8, 10)	<.001
Bloodwork days	6 (4, 8)	6 (4, 9)	.981
Scan days	1 (1, 2)	1 (1, 1)	.01
Additional outpatient visits	0 (0, 0)	0 (0, 0)	.665
Transfusion days	0 (0, 1)	0 (0, 2)	<.001
Hospitalization days	0 (0, 5)	11 (8, 17)	<.001

Data are median (interquartile range).

## Discussion

In this secondary analysis of the LY.12 trial, we demonstrate that measures of time spent in healthcare can be extracted from completed RCTs. In LY.12, despite broadly comparable short- and long-term oncologic outcomes, the GDP arm experienced fewer contact days. The sources of contact days also differed between arms, with the GDP arm spending relatively more days receiving outpatient chemotherapy, and the DHAP arm spending many more days in the hospital. Such information can add help patients and care partners understand expected time use, and guide decision-making for patients with aggressive hematological cancers, who already spend a significant portion of time with healthcare contact.

The primary finding of this work is that time spent undergoing treatment can be gleaned from secondary analyses of available data from previously published clinical trials. Our initial approach was to use clinical trial protocols and publications to reconstruct the course of the average patient on the trial arms, but this involved making several assumptions.^1^ The current work, and parallel work in the advanced solid tumor setting, establishes that patient-level analysis is feasible, especially when trials include pre-planned economic analyses. Cooperative group trials, that often collect resource utilization data, are well positioned to undertake such analyses. To prospectively assess contact days in future trials without further burdening clinical trial teams, a potential solution is linking trial and claims data. The LY.12 trial itself provides a successful example of this approach. Among 140 Canadian LY.12 trial participants across 9 provinces invited, 82% provided consent and identifying information to facilitate long-term administrative data linkage.^13^ We additionally selected the LY.12 trial for this analysis since efficacy outcomes between arms were similar—in such cases, the contact days measure may serve as a “’tie-breaker”’. Retrospective data should ideally not be used to compare the time use associated with different treatments due to residual confounding. However, due to difficulties in accessing RCT data, well-conducted real-world studies can help understand contact days with different approaches. In a single-institution study of 113 older adults with acute myeloid leukemia treated with azacytidine plus/ minus venetoclax at an academic U.S. center, with a median overall survival of 7.7 months, the average proportion of just oncology-related contact days was 42%, and did not differ by treatment approach.^14^

These data also provide elements of detail that can help improve care for vulnerable patients. First, over the 6-10 week salvage chemotherapy period, participants in both arms spent more than one in 3 days with healthcare contact. While patients often receive extensive logistic support during and after stem cell transplant, this is not always true during salvage regimens.^7,8^ Patients with relapsed/ refractory leukemia may spend even half the days during salvage chemotherapy hospitalized.^13^ These data serve as an urgent call to formally assess and address patient and care partner experiences during salvage chemotherapy. Second, while patients in the DHAP arm had slightly more contact days than patients in the GDP arm (38% vs 34%), the sources of contact days were quite different. Patients in the GDP arm who received primarily outpatient chemotherapy, as expected, had more outpatient chemotherapy days, while patients in the DHAP arm had much higher rates of hospital days (median 11 vs 0 days)—this reflects that DHAP was delivered in the hospital, and also that rates of physical toxicity requiring hospitalization were higher in the DHAP arm. Hospitalization days, in addition to likely representing days with physical toxicity and particularly “”low quality-of-life’’, also are a major contributor to health system costs.^10^ Our ongoing qualitative work assesses patient and care partner experiences with and perceptions of the relative burdens of different contact days (inpatient vs outpatient). While it is easy to assume that patients universally prefer outpatient days to inpatient days, repetitive and frequent outpatient visits can themselves impose significant logistic burdens, especially when travel distances are long and coordination of care is suboptimal.^15^ Some patients—especially those who live locally—are pleased to sleep in their own beds and spend hours every day in the hospital day unit. Others may prefer to stay in hospitals for logistical ease and a feeling of security. Thus, the contact days measure, and the sources of contact days, should be objectively presented to patients, who may have individual preferences and values.

This work has limitations. First, the current measure itself has limitations. It currently does not consider home-based care or telemedicine visits, and does not consider care partner time use. It is currently not quality-weighted. Ongoing qualitative work is addressing this. Second, the exact date of contact for a few outpatient visits was missing. We considered these visits to be on a separate day. We were unable to capture some trial-related contact days, eg, related to screening, prior to the date of assignment. These issues applied to both arms and should not affect comparisons.

In conclusion, we demonstrate that secondary analyses of a completed cooperative group trial that prospectively collected resource utilization data can be used to extract measures of time use. Data on patterns of time use in oncology clinical trial reports can supplement oncology efficacy data to aid patient-oncologist decision making, especially when differences in efficacy are small. This work is particularly relevant to people with hematologic malignancies who already face significant time with healthcare contact.

## Data Availability

The data underlying this article will be shared on reasonable request to the corresponding author.
